# Composition, Morphology, and Topography of Galvanic Coatings Fe-Co-W and Fe-Co-Mo

**DOI:** 10.1186/s11671-017-2128-3

**Published:** 2017-05-15

**Authors:** Iryna Yu. Yermolenko, Maryna V. Ved`, Nykolay D. Sakhnenko, Yulya I. Sachanova

**Affiliations:** National Technical University Kharkiv Polytechnical Institute, 2 Kyrpychova St., Kharkiv, 61002 Ukraine

**Keywords:** AFM analysis, Composition, Electrolyte bath, Fe-Co-W (Mo) coating, Pulse mode, Topography

## Abstract

Ternary coatings Fe-Co-W with an iron content of 40–55 at.%, cobalt 39–44 at.%, and tungsten 4–12 at.% and Fe-Co-Mo with an iron content of 40–55 at.%, cobalt 39–44 at.%, and tungsten 4–12 at.% were obtained by galvanostatic and pulse electrolysis on the mild steel substrate from iron(III) citrate-based electrolyte. The influence of electrolysis mode and parameters on composition of deposited alloys was studied. The competing reduction of iron and tungsten in Fe-Co-W coatings as well as the competitive deposition of iron and cobalt in Fe-Co-Mo coatings at various current densities were defined. Simultaneously, the alloy enrichment with molybdenum is more marked at a pulse mode. Atomic force microscope analysis of the Fe-Co-W alloy coating morphology and surface topography indicates their globular structure with spherical grains in the range of 2.5–3.5 μm. The surface of Fe-Co-Mo is characterized by parts of a globular structure with an average conglomerate size of 0.3–0.5 μm and singly located cone-shaped hills with a base diameter of 3 μm. Sites with a developed surface were detected within the same scan area which topography is identical to the crystal lattice of cobalt with the crystalline conglomerate sizes in the range of 0.2–1.75 μm.

## Background

The researchers’ and technologists’ increased interest to multicomponent galvanic alloys of iron triad metals with refractory components (W, Mo, and etc.) [1, 2] is caused by several reasons. The first is creation of new technology of coatings with a unique set of functional properties such as wear resistance and corrosion resistance, increased catalytic activity and microhardness, and magnetic properties [3, 4]. This allows to replace toxic chromium plating and to create effective catalytic materials, more available compared to traditional platinum-based systems [5, 6].

Secondly, the scientific interest is connected with the solution of theoretic problems determining the mechanism of “induced co-deposition” [7] and physical and chemical sense, of which is the conjugate electrochemical reduction of two or more metals. However, the mechanism of induced deposition is the subject of much debate up to date [8].

Obviously, in each individual case, the formation of the coating depends on the qualitative and quantitative composition of the electrolyte and on the synthesis conditions, wherein the modes and parameters of the electrolysis will determine in a particular way the concentration ratio of the alloy components and phase composition of the coatings [9].

The functional properties of the coatings are structurally dependent, so both their composition and surface morphology will determine the performance characteristics of materials. The roughness and surface friction are the main characteristics of the surface quality of the coatings. Previously, it was shown [10] that binary and ternary coatings obtained in a pulse mode are characterized by higher microhardness and wear resistance due to their smooth surface. The roughness of electrolytic deposits can be significantly decreased using pulse mode [11]. Since the structure of the electrolytic alloys determines both the properties and application of coatings, study of their morphology and topography remains relevant.

In spite of a sufficient number of works devoted to the binary alloy Fe (Co, Ni)-W (Mo) [12–14] patterns concerning the electrolysis mode influence on composition of ternary alloys, their topography and morphology require detailed investigation.

## Methods

The Fe-Co-W coatings were formed on a mild steel substrate and on a copper substrate from electrolytic bath of composition: (M) iron(III) sulfate 0.1–0.15, cobalt sulfate 0.15–0.2, sodium tungstate 0.04–0.06, sodium citrate 0.3–0.4, sodium sulfate 0.1, and boric acid 0.1; the pH value was adjusted within the range of 4.0–4.5 by addition of sulfuric acid or sodium hydroxide. The Fe-Co-Mo coatings were formed on a mild steel substrate and on a copper substrate from the same electrolyte but containing the sodium molybdate 0.06–0.08 M instead of sodium tungstate; the pH value was adjusted within the range of 3.5–4.5. Rectangular samples with a surface area of 2 × 10^−2^ dm^2^ were used as working electrodes.

Pretreatment of sample surface included mechanical polishing, polishing, degreasing, chemical etching in a mixture of 10% hydrochloric acid and 10% sulfuric acids, thorough washing with distilled water, and drying.

The coatings were formed in two modes: (i) galvanostatic with the current density *i* 2–6 A dm^−2^ and (ii) pulsed with unipolar pulse current with the amplitude *i* of 2–4 A dm^−2^ at a pulse duration *t*_on_ = 1°10^−2^–2°10^−2^ s and pause time *t*_off_ = 1°10^−2^–5°10^−2^ s. As anode served plates of AISI 304 steel; the cathode-to-anode area ratio was 1:5, volume current density was kept at the level 2 A dm^−3^. Both the galvanostatic and pulse electrolyses were performed using dc and pulse current supply unit (ZY-100±12).

The coating time was 20 to 30 min and deposit thickness was 8–10 μm according to the electrolysis time. The coatings with thickness of 30 μm were deposited onto copper substrate only for X-ray analysis.

The chemical composition of the coatings was determined by X-ray fluorescence method using a portable spectrometer “SPRUT” with a relative standard deviation of 10^−3^–10^−2^. The error at determining the content of the components is ±1 mass percent. To verify the results, the energy-dispersive X-ray spectroscopy was performed using an electron probe micro-analyzer Oxford INCA Energy 350 integrated into the SAM system. The content of components (in terms of metal) in the coatings are presented in at.%.

The structure of the coatings was examined by X-ray diffraction analysis using a diffractometer (DRON-2.0) in the emission of iron anode.

The surface morphology of Fe-Co-W and Fe-Co-Mo thin films was studied by an atomic force microscopy (AFM) using an NT–206 microscope. The tapping mode was conducted to measure samples’ surface morphologies. Scanning was performed by using the contact probe CSC-37 with a cantilever lateral resolution of 3 nm [15]. And the scan sizes were fixed at 39.9 × 39.9 μm and 10.0 × 10.0 μm, and the height of the surface relief was recorded at a resolution of 256 × 256 pixels. For each sample, a variety of scans were obtained at random locations on the surface of Fe-Co-W and Fe-Co-Mo thin films. In order to analyze the AFM images, all image data were converted into the Surface Explorer software. The root mean square (*R*_q_), mean particle height and its distribution, surface skewness, and particle diameter were obtained.

## Results and Discussion

Previous studies [16] have shown that co-deposition of iron and cobalt with molybdenum and tungsten may be held both in galvanostatic and pulse modes. The current density, electrolysis duration, and pulse and pause time will influence on the process efficiency, coating quality, and refractory component content in the deposit.

**Fig. 1:**
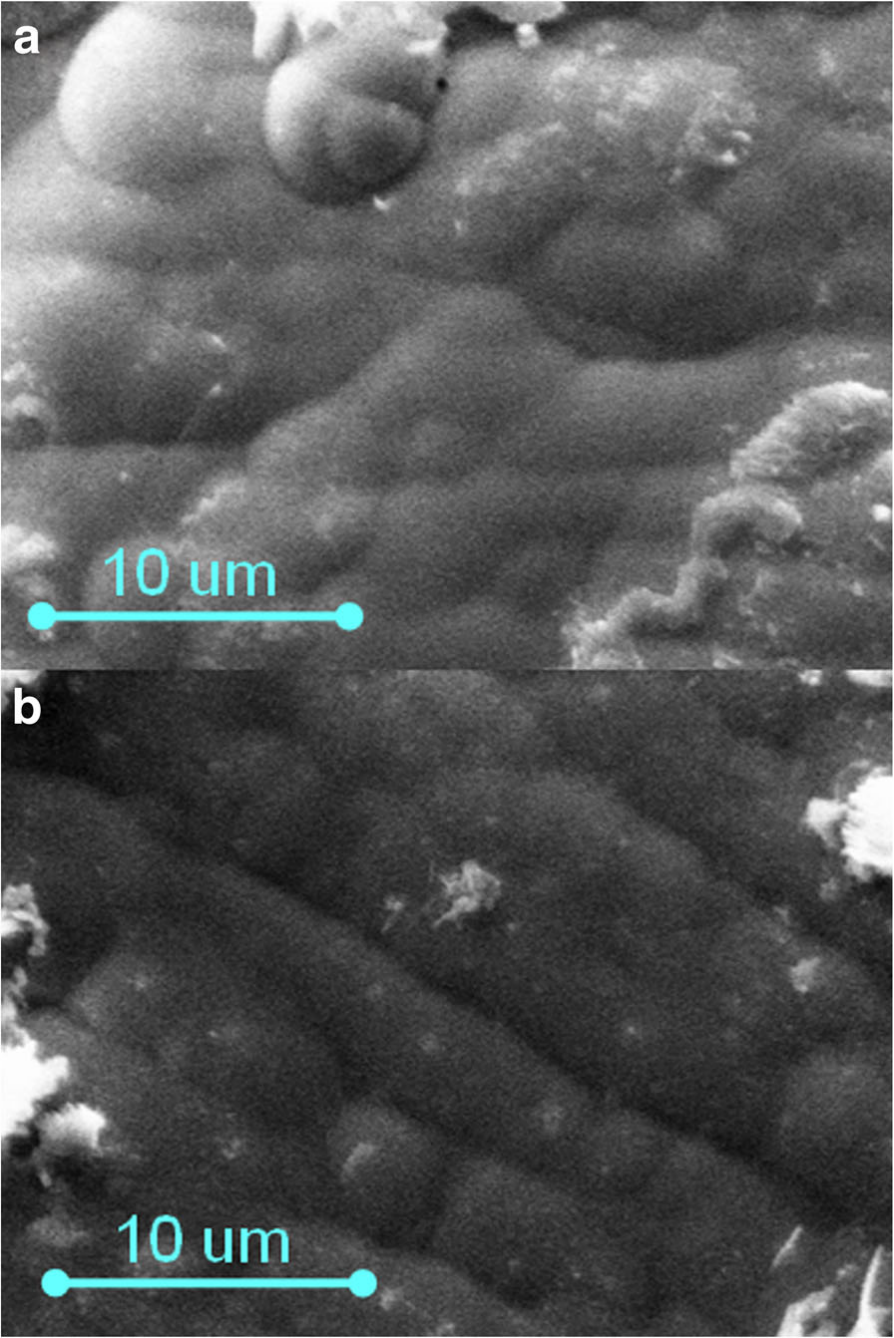
SEM images of Fe-Co-W coatings of thickness 10 μm deposited onto copper substrate. **a** Galvanostatic electrolysis, *i* = 3 A dm^−2^. **b** Pulse mode, *i* = 3 A dm^−2^, *t*_on_/*t*_off_ = 20 ms/20 ms

The matte Fe-Co-W coatings with an iron content of 48–55 at.%, cobalt 39–42 at.%, and tungsten 4–12 at.%. (Fig. 1a) were obtained on the mild steel substrate by a galvanostatic mode. The composition of electrolytic alloys deposited using a unipolar pulsed current is 40–53 at.%, cobalt 40–44 at.%, and tungsten 7–10 at.% (Fig. 1b). Globular surface of Fe-Co-Mo coatings deposited using a direct current is caused by the presence of the refractory component [17], and its unevenness is greater than that of the coating deposited in a pulse mode.

**Fig. 2:**
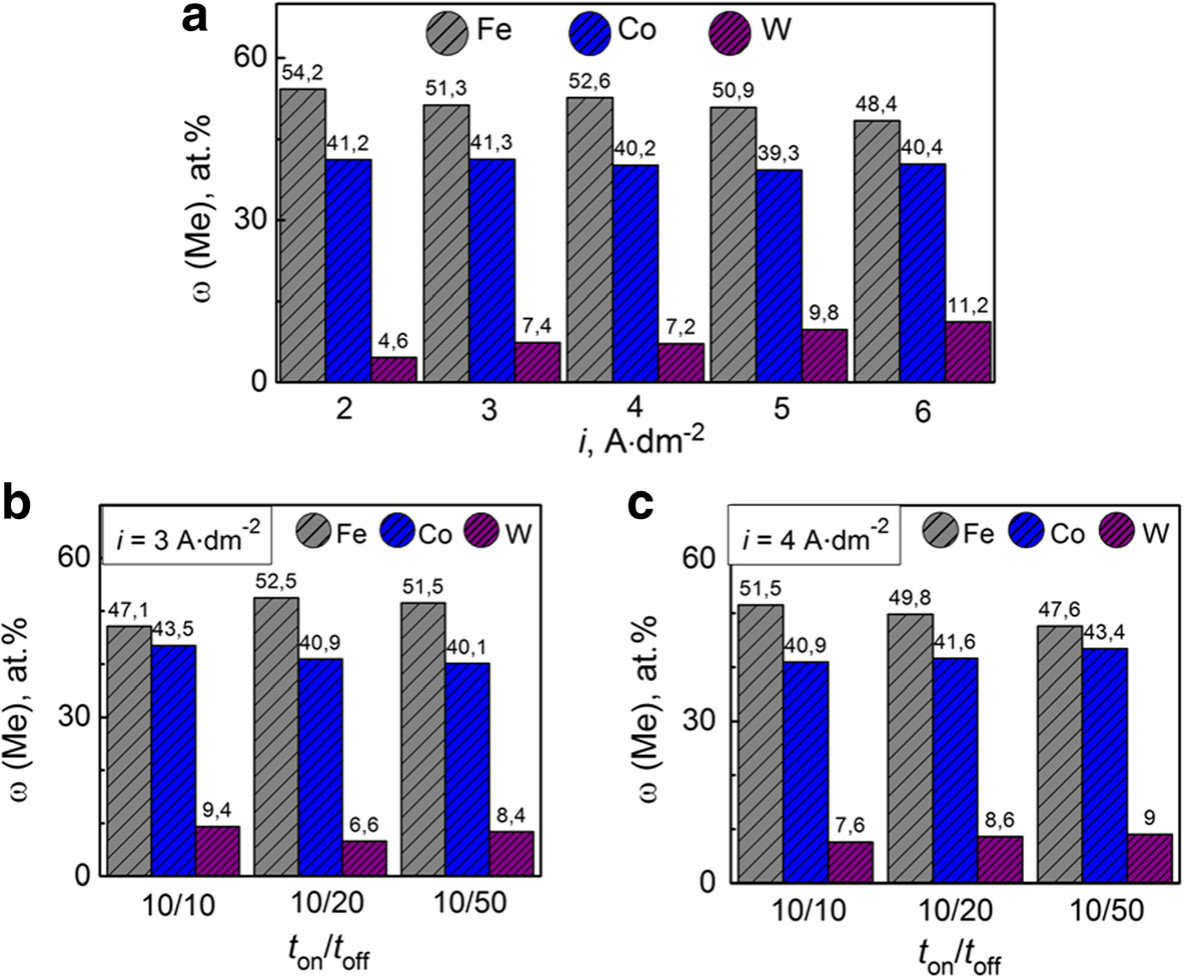
Current density influence on the composition of Fe-Co-W alloys with thickness of 10 μm deposited onto copper substrate. **a** Galvanostatic electrolysis. **b**, **c** Pulse mode

The iron and tungsten competitive deposition in the alloy Fe-Co-W (Fig. 2) is observed in a galvanostatic mode at a varying current density of 2–6 A dm^−2^. The tungsten content in the coating increases from 4 to 11 at.% by reducing the iron content with increasing current density from 2 to 6 A dm^−2^ as one can see from Fig. 2a. This is due to the intensification of parallel reaction of hydrogen reduction forming ad-atoms H_ad_, which contributes to a more complete chemical reduction of intermediate tungsten oxides. The content of cobalt in this case varies within the range of 2 at.%. The maximum current efficiency is defined as 40% at a current density of 4 A dm^−2^. Increasing the current density up to 6 A dm^−2^ leads to a decrease of current efficiency to 27% caused by the contribution of hydrogen evolution reaction into the overall cathode process.

Unipolar pulsed current significantly increases the efficiency of the cathode process, which is related to more complete implementation of reactant adsorption, product desorption, chemical reduction of intermediate tungsten oxides by hydrogen ad-atoms, and ligand removal in a pause period.

The current efficiency increases almost twice compared with a stationary mode and is 70–75 and 63–68% at a current density of 3 and 4 A dm^−2^ respectively.

The content of the refractory component in the coatings is higher than in the deposits obtained in the galvanostatic mode at similar current densities as can be seen from Fig. 2b, c.

Increasing pause duration at a current density of 4 A dm^−2^ and pulse duration of 10 ms leads to competitive reduction of cobalt and tungsten with iron decreasing the content of the latter (Fig. 2c).

Varying the ratio of the pulse/pause allows to precipitate coating with extended range of alloy components and therefore with a different level of functional properties. This will significantly expand the scope of the ternary alloy Fe-Co-W.

**Fig. 3:**
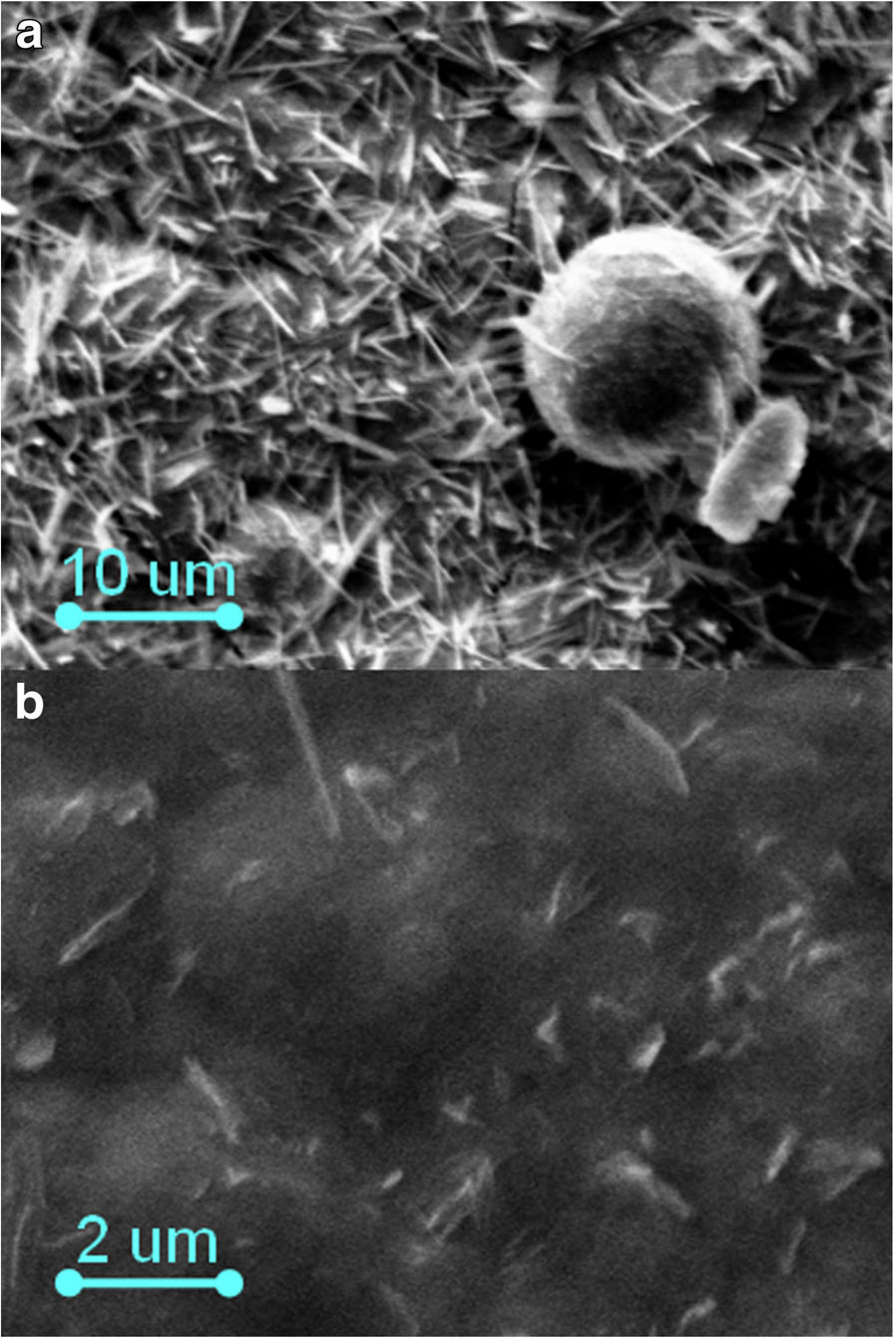
SEM images of Fe-Co-Mo coatings with thickness of 10 μm deposited onto copper substrate. **a** Galvanostatic electrolysis, *i* = 3 A dm^−2^. **b** Pulse mode, *i* = 2 A dm^−2^, *t*_on_/*t*_off_ = 20 ms/20 ms

Fe-Co-Mo coatings are characterized by significantly different relief of deposits obtained in different electrolysis modes (Fig. 3). The coatings with an iron content of 49–53 at.%, cobalt 36–39 at.%, and molybdenum 11–13 at.% were deposited in a stationary mode. The fine crystalline deposits are formed at a current density of 2.5–3.0 A dm^−2^, and one can see individual spheroids at their surface (Fig. 3a).

On the contrary, more uniform deposits with globular structure with singly located crystallites are formed by the non-stationary mode (Fig. 3b).

**Fig. 4:**
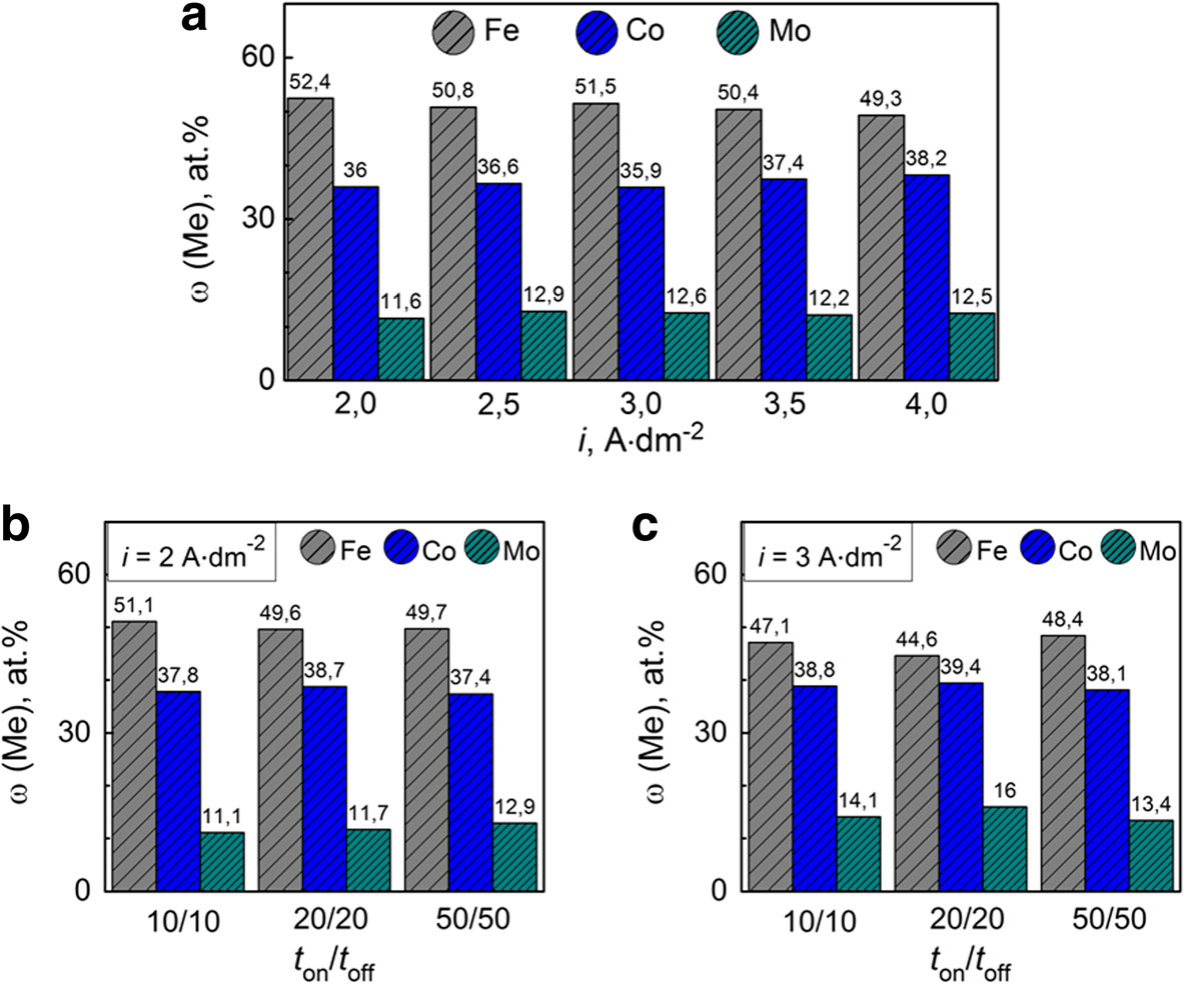
Current density influence on the composition of Fe-Co-Mo alloys (thickness 10 μm) deposited onto copper substrate. **a** Galvanostatic electrolysis. **b**, **c** Pulse mode

The results of elemental analysis of Fe-Co-Mo coatings obtained on a substrate of mild steel at various current densities demonstrate competition process of recovery of iron and cobalt. Increasing current density increases the cobalt content of the alloy; the iron content thus decreases (Fig. 4a). The intensification of hydrogen evolution reaction takes place at high current densities as a result when alkalinization is near the electrode layer. This leads to the formation of iron(II) and iron(III) hydroxy compounds (*pК*_in_ [Fe(OH)^+^] = 5.56; *pК*_in_ [Fe(OH)^2+^] = 11.8) with higher stability than cobalt hydroxy particles (*pК*_in_ [Co(OH)^+^] = 4.4) which contributes to cobalt reduction in the coating. As a result, the cobalt content in the alloy increases. Wherein the molybdenum content ranges from 2 at.%, the maximum molybdenum content of 13 at.% is reached at a current density of 3 A dm^−2^ (Fig. 4a).

It should be noted that the current efficiency for Fe-Co-Mo coating deposition in the galvanostatic mode is higher than for the Fe-Co-W alloy and is in the range of 43–65%.

The unipolar pulse current provides a Fe-Co-Mo coating with a wider range of alloy components. The iron content in the coating varies in the range of 44–52 at.%, cobalt 37–40 at.%, and molybdenum 11–16 at.%. The trend to the iron content decreasing accompanied with some cobalt content increasing is saved with rising pulse current amplitude, as seen from Fig. 4b, c. Enrichment alloy with molybdenum is observed simultaneously to the competitive reduction of iron and cobalt. The current efficiency *C*_e_ of alloy deposition at a current density of 3 A dm^−2^ is of 45–60% and is lower as compared to *C*_e_ = 70–82% for coatings obtained at *i* = 2 A dm^−2^.

Intensification of competitive reduction of the alloying metals at varying the time parameters of pulsed electrolysis (on time *t*_on_, ratio on/off time *t*_on_/*t*_off_) should also be noted.

Analysis of the results shows that ternary alloys Fe-Co-W and Fe-Co-Mo deposition by a unipolar pulse mode is more effective and allows to obtain coatings with target content of the components in the alloy.

**Fig. 5:**
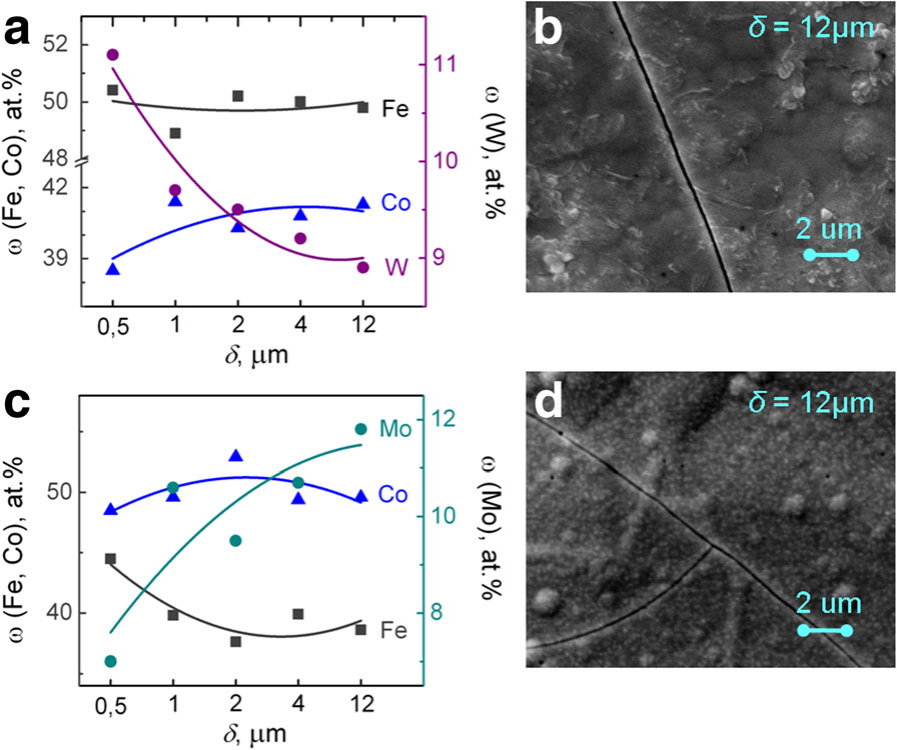
The distribution of alloying elements over the thickness of the coating and SEM images of coatings deposited in galvanostatic mode. **a**, **b** Fe-Co-W. **c**, **d** Fe-Co-Mo

The distribution of alloy-forming metals over the thickness of the coating is studied using a layer-by-layer deposition. It was found that the tungsten content in the deposit Fe-Co-W decreases in the direction from the substrate to the surface of the coating (Fig. 5a). The surface of the coating Fe-Co-Mo on the contrary is enriched by molybdenum in comparison with the inner layers (Fig. 5c). In addition, there is a tendency to cracking both coatings with an increase in the coating thickness of more than 10 μm (Fig. 5b, d).

**Fig. 6:**
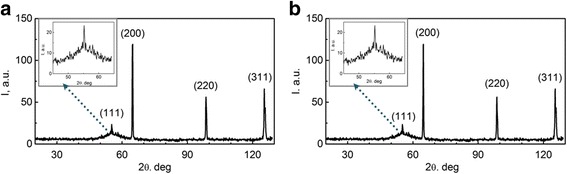
X-ray diffraction patterns of **a** Fe-Co-W and **b** Fe-Co-Mo coatings with thickness of 30 μm on copper substrate

The structure of the coatings Fe-Co-W with an iron content of 47 at.%, cobalt 43 at.%, and tungsten 10 at.% (in terms of metal) (Fig. 6a) and Fe-Co-Mo with an iron content of 50 at.%, cobalt 39 at.%, and molybdenum 13 at.% (Fig. 6b) that was formed on a copper substrate with a thickness of 30 μm was examined. The lines of the copper substrate are present on both diffraction patterns. As can be seen from Fig. 6, diffraction patterns for the alloys Fe-Co-W and Fe-Co-Mo are identical and are characterized by low halo at angles 2θ 55°–65°. This indicates that alloys are X-ray amorphous.

Traditionally, in materials science, the roughness is an indicator of surface quality and depends on the material processing. The roughness of galvanic coatings is the result of the alloy deposition and may serve as an additional indicator of the surface development as well as topography [18].

The coating samples Fe-Co-W and Fe-Co-Mo containing refractory component of 10–12 at.% obtained on mild steel were used for AFM analysis.

**Fig. 7:**
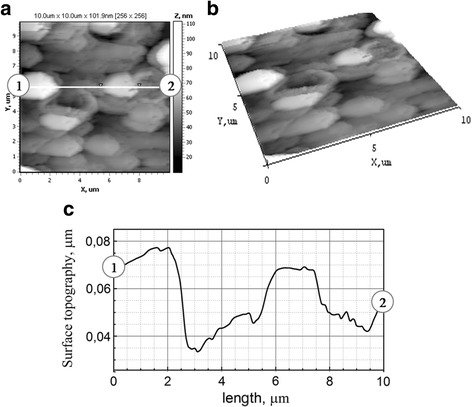
**a** 2D and **b** 3D maps of the surface and **c** cross-sections between markers 1 and 2 for mild steel substrate. Scan area AFM 10.0 × 10.0 μm

The substrate of mild steel is characterized by an even surface (Fig. 7a, b) with roughness *R*_a_ = 0.007 and *R*_q_ = 0.010. However, the structure of the surface is not ordered. The cross-section of profile between markers 1 and 2 indicates that the grain sizes are in the range of 2–3 μm as one can see from Fig. 7c.

**Fig. 8:**
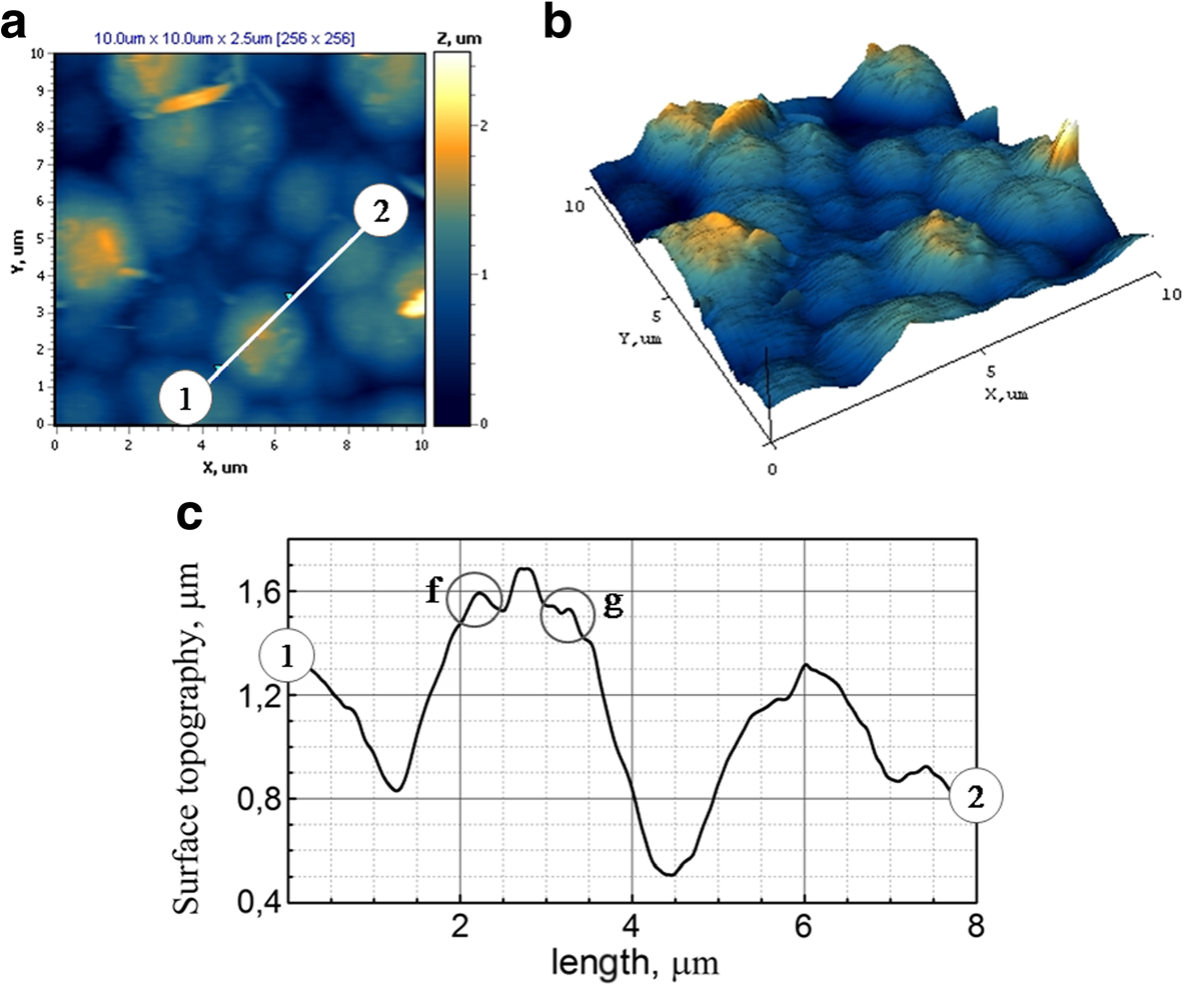
**a** 2D and **b** 3D maps of the surface and **c** cross-sections between markers 1 and 2 for Fe-Co-W coatings with thickness of 10 μm deposited onto mild steel substrate. Scan area AFM 10.0 × 10.0 μm

The surface of Fe-Co-W alloy is characterized by a more ordered structure than the substrate and the presence of agglomerates of spherical grains (Fig. 8a, b). The cross-section of profile between markers 1 and 2 indicates that the agglomerate sizes are in the range of 2.5–3.5 μm, wherein the surface of larger spheroids is formed with a smaller grain size of 0.3–0.5 μm as one can see from Fig. 8c for fields “f” and “g.”

The parameters *R*_a_ and *R*_q_ for Fe-Co-W alloy were defined as 0.3 which is much higher than those for the substrate and shows substantial development of the surface.

It was established earlier [19] that globular structure of the surface is caused by the refractory metals present in the alloy. We can expect the increased microhardness and catalytic properties of the resulting Fe-Co-W coatings in this case as shown in the results of previous researchers [20].

**Fig. 9:**
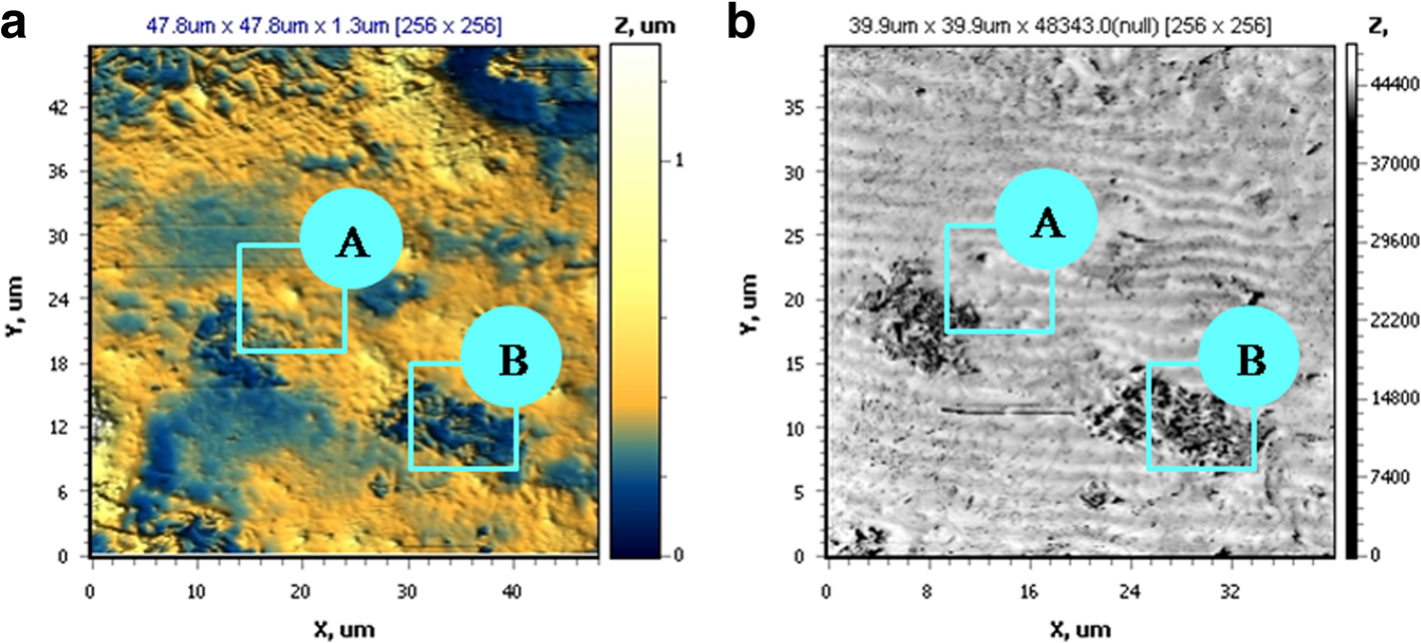
**a** 2D topography and **b** 2D torsion maps of the surface for Fe-Co-Mo coating on mild steel substrate with thickness of 10 μm deposited in pulse mode

While the surface of Fe-Co-Mo coatings (scanning area 39.9 × 39.9 μm) is more developed and globular comparing with the substrate as it follows from AFM 2D map analysis (Fig. 9), moreover, one can observe the parts of different morphologies—site A and site B—at the surface of Fe-Co-Mo cover (Fig. 9a, b).

**Fig. 10:**
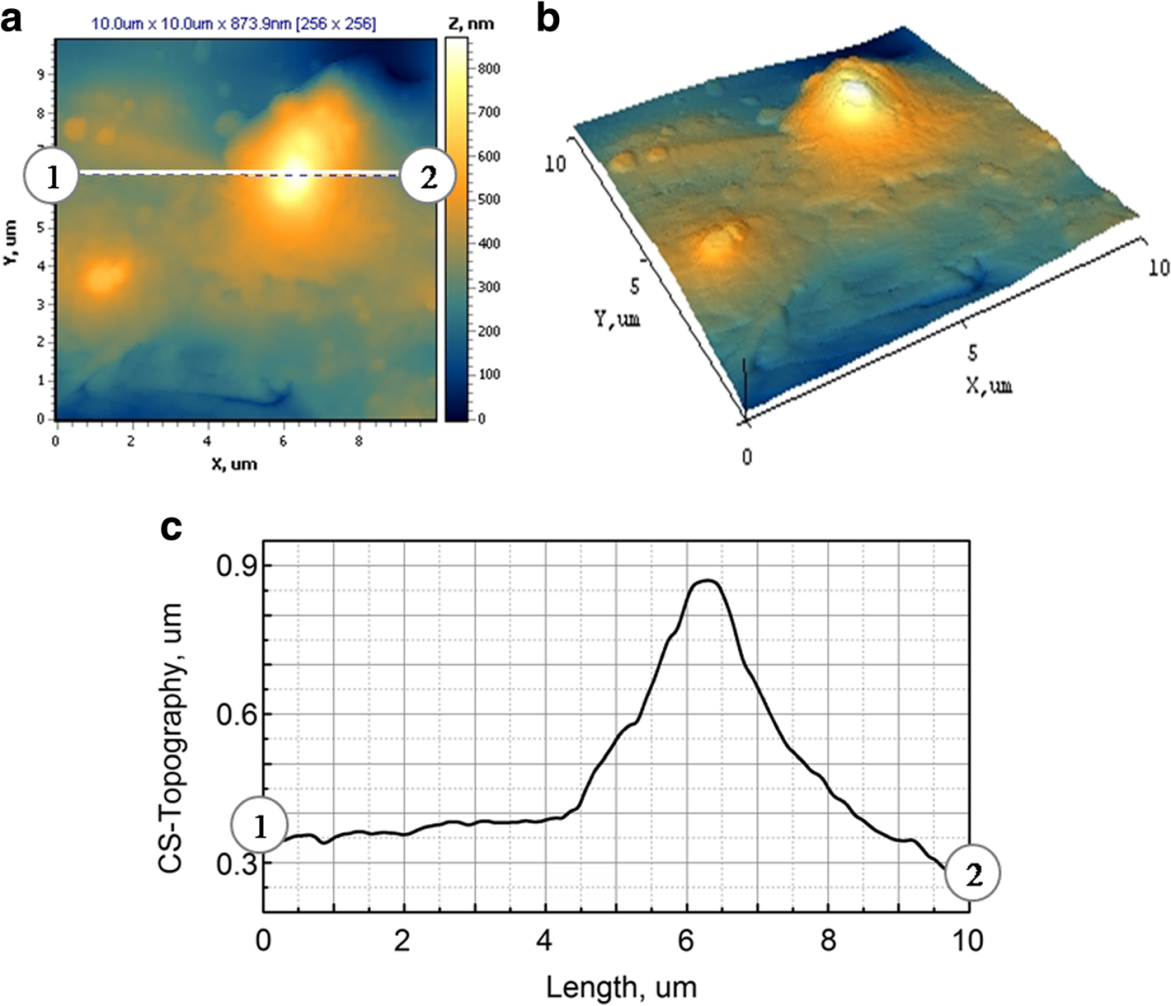
**a** 2D and **b** 3D maps of the surface and **c** cross-sections between markers 1 and 2 for part A of Fe-Co-Mo coating with thickness of 10 μm deposited onto mild steel substrate in pulse mode. Scan area AFM 10.0 × 10.0 μm

**Fig. 11:**
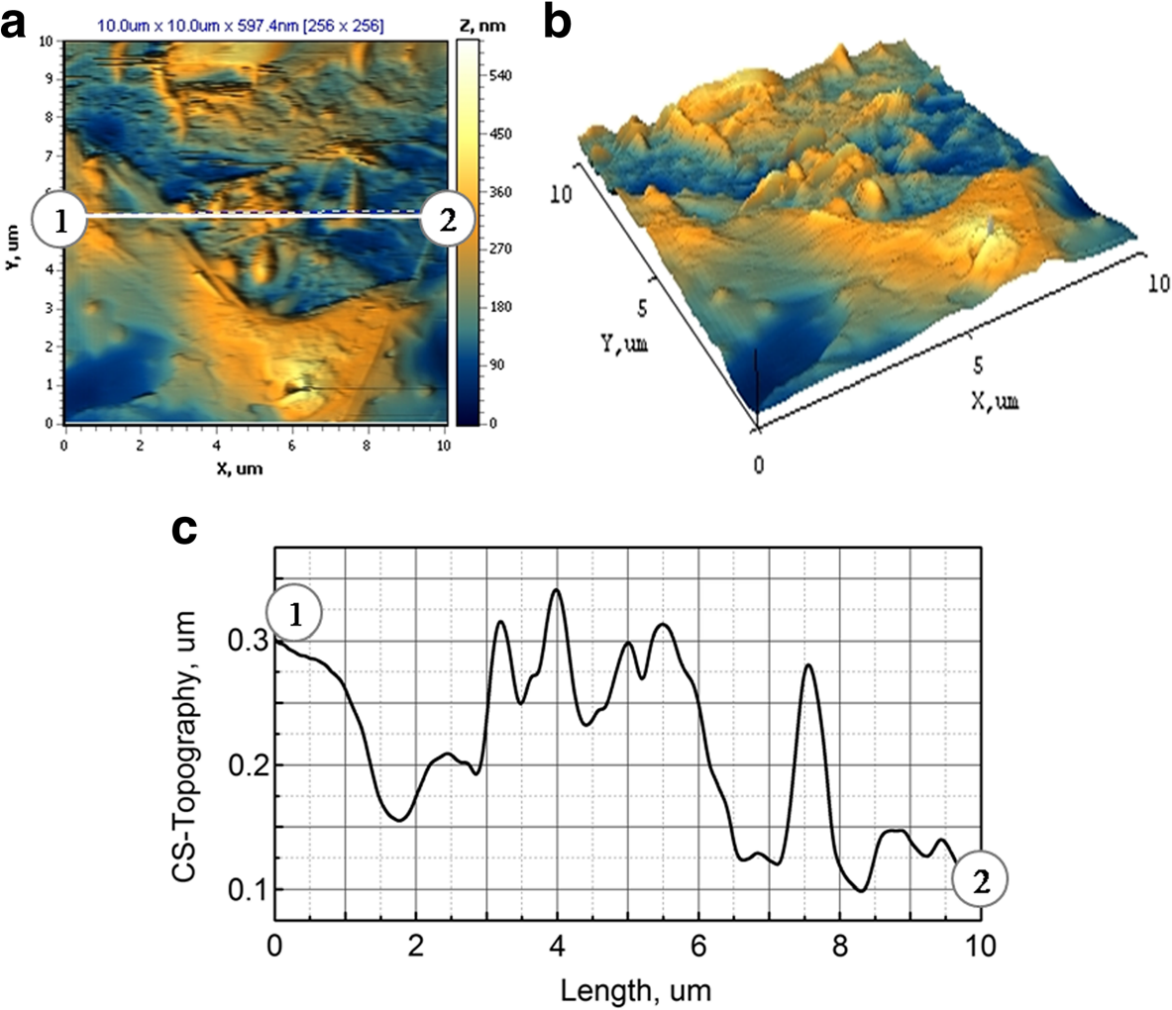
**a** 2D and **b** 3D maps of the surface and **c** cross-sections between markers 1 and 2 for part B of Fe-Co-Mo coating with thickness of 10 μm deposited onto mild steel substrate in pulse mode. Scan area AFM 10.0 × 10.0 μm

The torsion of measuring console in one scan area has a substantial change in skin friction at abovementioned parts (Fig. 9b), indicating that the surface of the material is non-homogeneous [21, 22]. In this regard, the more detailed study of the morphology of parts A (Fig. 10) and B (Fig. 11) is of great interest and importance.

Part A is characterized by the even structure with an average size of conglomerates 0.3–0.5 μm and singly located cone-shaped hills with a base diameter of ~3 μm and a height of 0.6 μm as one can see from Fig. 10c. As appears from 2D and 3D map topography of the surface (Fig. 10a, b), the cone-shaped hills are formed of the smaller spheroids.

Site B is characterized by more developed surface compared with site A. The hexagonal crystal lattice of cobalt with sufficiently sharp hills alternating by valleys is visualized at the 2D and 3D maps of the coatings’ surface (Fig. 11a, b). The cross-section of profile between markers 1 and 2 indicates that the crystalline conglomerate sizes are in the range of 2.0–4.0 μm, wherein the surface of larger crystalline size of 2.0–4.0 μm is formed with a smaller grain size of 0.5–1.0 μm as one can see from Fig. 11c.

The parameter *R*_q_ for part A and part B was defined as 0.35 and 0.30, respectively, reflecting the greater roughness of part A caused by availability of the high hills. However, values of *R*_q_ for parts of different morphology have no significant effect on the average roughness of the coatings *R*_a_ = 0.25. Accordingly, to the *R*_a_ and *R*_q_, the Fe-Co-Mo coating has a roughness class surface of 8–9.

## Conclusions

1. The ternary coatings Fe-Co-W obtained by galvanostatic and pulse electrolysis modes are characterized by a more ordered structure than the substrate and the presence of agglomerates of spherical grains in the range of 2.5–3.5 μm. The results indicate the competing processes of recovery of iron and tungsten when forming Fe-Co-W coatings.
2. The ternary coatings Fe-Co-Mo obtained by galvanostatic and pulse electrolysis modes are characterized by developed surface containing sites with globular structure and hexagonal crystalline conglomerate sizes in the range of 2.0–4.0 μm lattice of cobalt. The results of elemental analysis of Fe-Co-Mo coatings obtained on a substrate of mild steel 08KP at various current densities demonstrate competition process of recovery of iron and cobalt.
3. The unipolar pulse mode is more effective and allows to obtain coatings with the specified content of the components in the alloy at the electrodeposition of ternary alloys Fe-Co-W and Fe-Co-Mo.
4. Synthesized Fe-Co-W and Fe-Co-Mo coatings with average roughness of 0.25 can be attributed to 8th to 9th roughness class.
